# Situs Inversus Totalis in the Neonatal Setting

**DOI:** 10.7759/cureus.13516

**Published:** 2021-02-23

**Authors:** Jordan Devera, Francesca Licandro, Jean Ramos, Hovik T Taymoorian, Laurel G Yap

**Affiliations:** 1 Obstetrics and Gynecology, Pediatrics, and Medicine, University of Medicine and Health Sciences, Camps, Basseterre, KNA; 2 Obstetrics and Gynecology, MedStar Harbor Hospital, Baltimore, USA; 3 Neonatology, MedStar Harbor Hospital, Baltimore, USA

**Keywords:** situs inversus with dextrocardia, situs inversus totalis, primary ciliary dyskinesia, kartagener syndrome

## Abstract

Situs inversus totalis (SIT) is a rare condition of complete inversion and mirroring of normal human anatomy. The incidence is approximately 1 in 8,000 to 1 in 25,000 live births. SIT is inherited in an autosomal recessive pattern and is associated with multiple gene mutations. It is also commonly seen in a condition known as primary ciliary dyskinesia.

A 39-year-old pregnant woman presented to the Labor and Delivery unit to rule out pre-eclampsia due to high blood pressure recordings in the office setting. The infant was delivered preterm at 36 weeks gestation via spontaneous vaginal delivery. The infant presented with symptoms of respiratory distress. The newborn was transferred to the neonatal intensive care unit (NICU) for further work-up and to rule in/rule out an etiology known as Wet Lung. Upon retrieving a chest X-ray for the newborn, the results demonstrated situs inversus totalis. The newborn was transferred to a level III NICU for further management and work-up for other potential etiologies. Situs inversus totalis was not seen on prenatal work-up.

In summary, situs inversus totalis is a rare condition which can be associated with other detrimental conditions. In the future, if situs inversus totalis is detected in utero, patients should be instructed to deliver in a setting in which any possible etiology can be accommodated. Pediatricians should follow these infants closely and with caution as common presentations may be obscured due to complete inversion of normal human anatomy. It is also important to screen these infants for other etiologies which may present in later developmental stages such as bronchiectasis and respiratory infections.

## Introduction

Situs inversus totalis (SIT) is a rare condition that is defined by the complete positional inversion of cardiac and abdominal viscera. The normal position of the heart is seen with the apex pointing toward the left aspect of the body. However, in situs inversus totalis, the apex points towards the right due to the positional mirroring of the normal anatomy. This is also termed situs inversus with dextrocardia. Situs inversus is associated with an autosomal recessive pattern of inheritance. The absolute cause of situs inversus totalis is unknown, but in 2002, Bartoloni et al. studied a mutation in the gene DNAH11 that seems to account for one form of situs inversus [1]. In addition, other genetic components that have shown an association with the condition are lefty genes, nodal genes, and ZIC 3, ACVR2B and Pitxz genes [2]. The following case report describes a case of situs inversus totalis which was unknown in utero and was discovered incidentally in the neonate setting.

## Case presentation

A 39-year-old woman, G4P0030 at 36 weeks and two days, with uneventful and well followed prenatal care, presented to the Labor and Delivery unit to rule out pre-eclampsia due to high blood pressure recordings in the office setting. The patient had a urine protein of 324 mg/24 hours and a blood pressure of 153/93 mmHg. She was subsequently diagnosed with mild pre-eclampsia. Following this diagnosis, the patient was given a dose of betamethasone to ensure fetal lung maturity at the time of delivery. The patient had negative viral serologies at this time.

**Figure 1:**
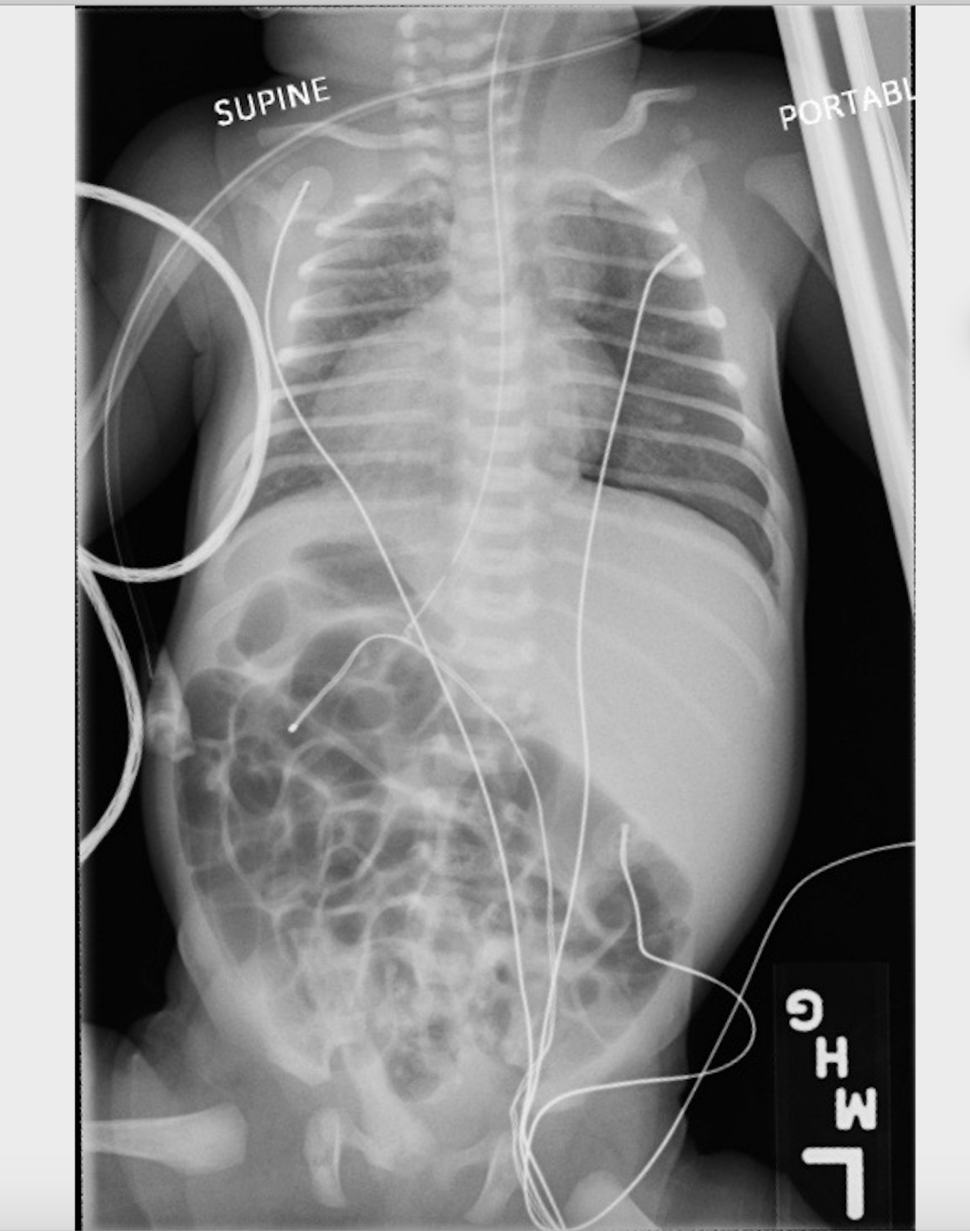
Chest X-ray of the infant suggesting situs inversus totalis

The woman proceeded to give birth to a single live preterm infant via spontaneous vaginal delivery. The infant presented with mild grunting, subcostal retractions, and nasal flaring. Accordingly, the neonate was given an APGAR score of 8 and 9. The infant’s lung sounds were clear and symmetric to auscultation; no crackles, rhonchi, or wheezing were noted. The remainder of the physical examination was within normal limits. The infant was active, reactive, pink, well perfused with no lesions, rashes, or deformities were noted. The infant had normal cardiac sounds with no crackles or rhonchi were noted. The femoral pulses were also palpated. The infant was transferred to the neonatal intensive care unit (NICU) for further observation and was started on oxygen support at a 3L flow. A chest X-ray was ordered to rule in/rule out a common etiology seen among newborn infants - Wet Lung. The results demonstrated that the apex of the heart was found in the right hemithorax, the stomach was noted to be in the right upper quadrant of the abdomen and the liver was found in the left upper quadrant of the abdomen. These findings are suggestive of dextrocardia with situs inversus. A follow-up chest X-ray was done in order to ensure the correct placement of the left and right markers. The follow-up chest X-ray confirmed the previous findings of dextrocardia with situs inversus totalis (Figure 1). The findings also demonstrated an opacity in the left superior mediastinum which could be secondary to the rotation of the patient and the thymic shadow. Preceding these findings, the infant was transferred to a level III NICU for further observation and work-up regarding this rare etiology.

## Discussion

Situs inversus totalis is an uncommon condition that has an incidence rate of 1/8,000 to 1/25,000 in live births [3]. An individual with situs inversus can have a normal uncomplicated life span but must be cautious when undergoing surgical procedures due to unfamiliar anatomical position of structures [4]. Situs inversus is closely associated with another condition termed primary ciliary dyskinesia (PCD). In this condition, the cilia are not functioning adequately which leads to recurrent respiratory infections and bronchiectasis seen in infancy that can display notable lung damage in adulthood. In addition, males may suffer from infertility secondary to sperm dyskinesia. Fifty percent of all PCD cases are associated with situs inversus which is defined together as Kartagener syndrome [1,5]. Situs inversus with dextrocardia is also commonly accompanied by the following congenital cardiac anomalies: atrial situs solitus, discordant atrioventricular (AV) connection, discordant ventriculoatrial (VA) connection, and to a lesser extent congenitally corrected transposition of great arteries (TGA) [6].

Our patient with situs inversus totalis was not diagnosed with this condition until in the neonatal setting. It was found on the investigation of respiratory distress in the preterm infant with good APGAR scores of 8 and 9. On chest X-ray to rule out/rule in Wet Lung, the first finding was of situs inversus totalis in this patient. This is important to note because the mother attended prenatal classes and was followed medically during pregnancy. Having the diagnosis of situs inversus totalis is an important finding because even if the patient does not have any congenital anomalies, which may indicate a normal life expectancy, the patient’s presentation of common ailments may become difficult to diagnose due to mirrored anatomy [7]. For example, if a patient with unknown sinus inversus totalis had appendicitis, most clinicians would rule it out based on location alone, which could lead to a detrimental outcome.

In our patient and other patients with situs inversus, it is imperative to observe symptoms and signs of recurrent respiratory infections and bronchiectasis. If present, it may indicate Kartagener syndrome. Therefore, their pediatricians should frequently monitor and follow these patients regularly. This infant was born at a hospital with a level II NICU due to situs inversus not being detected during prenatal visits. The infant was subsequently transferred to a level III NICU. In the future, it is important to discover anatomical variances in-utero in order to deliver the infant in an appropriate setting in which a higher level of intervention and greater resources may be available if indicated.

## Conclusions

Situs inversus is a rare condition that occurs in 1/8,000 to 1/25,000 individuals. Despite not knowing the complete cause of situs inversus totalis, it is essential to identify, monitor, and treat patients according to the degree of their condition. Many patients with situs inversus will have a normal life span but will need to inform their clinicians of their anatomical mirroring to prevent complications during interventions. For example, a surgeon must be aware of reversed anatomy during trochar placement in laparoscopic surgery. However, individuals suffering from situs inversus totalis with associated conditions may require a higher-level of intervention, which is why in utero identification is of notable importance.
